# Effects of water-soluble carbohydrates from grasses on the nutrition of grazing ruminants

**DOI:** 10.3168/jdsc.2025-0918

**Published:** 2026-02-19

**Authors:** M.A. Porcu, A. Cannas

**Affiliations:** Department of Agricultural Sciences, University of Sassari, Sassari 07100 Italy

## Abstract

Grasses used for grazing, particularly modern ryegrass varieties and other C3 grasses, show high variability in water-soluble carbohydrate (WSC) concentrations (5%–40% of DM) depending on species and varieties, environment, and management. High WSC concentrations, typical of high-latitude regions, have recently also been reported under Mediterranean conditions. In ruminants, pasture WSC may be positively associated with grazing time, DMI, animal performance, and pasture nutrient use efficiency. However, along with other nutritional factors, they can also be associated with rapid ruminal fermentation and low rumen pH, which may negatively affect milk fat synthesis under certain grazing conditions.

## Highlights

• WSC concentration is highly variable in grasses.
• C3 grasses can have high concentrations of WSC.
• WSC can be associated with rumen fermentation alterations.
• WSC of grasses may contribute to milk fat reduction.
• Optimal water-soluble carbohydrate level in ruminant diets is not well defined.

## Graphical Abstract

Water-soluble carbohydrates (WSC) consist of simple sugars (mainly glucose, fructose, and sucrose), oligosaccharides, and total fructans. Fructans are a distinct group of complex carbohydrates that accumulate through a non-self-limiting process, being the primary storage form of carbohydrates of cool-season (C3) grasses. The WSC concentrations in C3 grasses display diurnal and seasonal fluctuations, and are strongly affected by environmental conditions, plant part considered, growth stage, species, cultivar, and ploidy. The positive and negative effects of WSC intake on grazing ruminants are discussed, including possible associations with the reduction of ruminal pH and milk fat concentration. VFA = volatile fatty acids.

**Figure:**
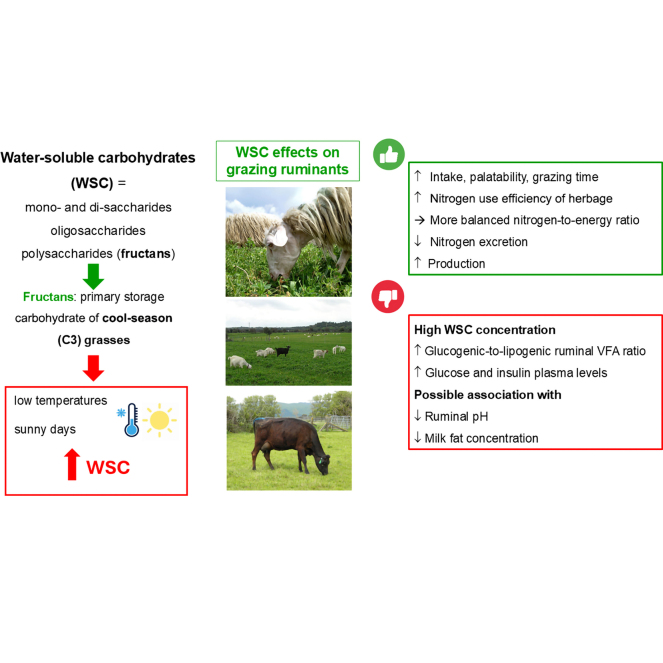

Ruminants often graze on pastures of cool-season grasses, namely temperate or C3 grasses. Cool-season grasses contain water-soluble carbohydrates (**WSC**), which are composed of simple sugars (monosaccharides, i.e., glucose and fructose; and disaccharides, i.e., sucrose) and complex carbohydrates (oligosaccharides and polysaccharides, i.e., fructans). In grasses, WSC can reach high concentrations under certain conditions and, thus, might have marked nutritional effects on grazing ruminants. Despite this, the literature on these issues is limited. Analytical methods for measuring these carbohydrates in feeds are numerous, not standardized, and differ among laboratories (Watts, 2004). Thus, the terminology used for the same carbohydrate fractions is variable and makes comparisons complicated (Geor, 2009). In feeds, these carbohydrates can be analyzed as WSC or ethanol-soluble carbohydrates (**ESC**).

The WSC are extracted with water (cold, at room temperature, or hot, depending on the method) and comprise glucose, fructose, sucrose, oligosaccharides, and the entire amount of fructans present in the grass (Klevenhusen and Zebeli, 2021; Kagan, 2022). In contrast, the ESC are extracted with 80% ethanol and comprise glucose, fructose, sucrose, oligosaccharides, and only short-chain fructans (Kagan et al., 2018; Klevenhusen and Zebeli, 2021). Fructans, which are a heterogeneous class of complex carbohydrates, are the prevailing WSC component in C3 grasses (Pollock and Cairns, 1991). The features of fructans vary among grass species (Geor, 2009). For instance, ryegrass fructans have low molecular weight, short chain length, and degree of polymerization (**DP**) of 30 to 40 units, whereas timothy and orchard grass contain larger fructans, with a DP equal to or greater than 100 units (Geor, 2009). Fructans are the primary storage carbohydrate in the leaves and stems of C3 grasses and Compositae, whereas starch, another polysaccharide, is the primary storage carbohydrate in warm-season (C4) grasses and legumes (Van Soest, 1994). In C3 grasses, fructan accumulation is not self-limiting (Longland and Byrd, 2006) and occurs in the cell vacuoles of leaf cells (Pollock and Cairns, 1991), with stems being their primary storage site (Watts, 2004). The concentration of WSC in C3 grasses is highly dynamic, reflecting the balance between photosynthesis, respiration, and their use for growth and development. It varies diurnally, with an afternoon peak and an overnight decline (Geor, 2009) through respiration. Numerous factors affect WSC accumulation, including plant part, growth stage, species, cultivar, ploidy, time of day, temperature, light intensity, season, water availability, fertilization, and crop management (Olszewska, 2021; Kagan, 2022). Stress conditions such as low temperatures, frost, drought, and low soil fertility (Geor, 2009) can result in WSC accumulation. During slow growth periods, photosynthetic products often exceed plant demand and large quantities of fructans accumulate in C3 grasses (Livingston et al., 2009).

The WSC accumulation in grass is also closely linked to weather conditions, increasing during winter–early spring, characterized by low temperatures, sunny days, and cold nights (Watts, 2005). Under these conditions, respiration rates decrease when temperatures are below 5°C (Watts, 2004), and plant growth may decrease or cease at freezing temperatures, although WSC continues to be synthesized and accumulated (Watts, 2005). In contrast, reduced solar radiation and sunlight hours in autumn reduce photosynthesis, whereas high night temperatures favor WSC respiration (Jafari, 2012). Indeed, WSC concentrations in grasses are lower in autumn than in winter–early spring (Rivero et al., 2020). Satta et al. (2023) reported this pattern in Mediterranean conditions on different grass species, with lower WSC concentrations in autumn (mean 3.5%, maximum 6.4% of DM) than in winter–early spring (mean 21.5%, maximum 36.2% of DM). Favorable conditions for plant growth typically reduce WSC concentration (Longland and Byrd, 2006). For example, increased nitrogen fertilization is inversely related to plant WSC concentration (Olszewska, 2021). After defoliation by grazing or cutting, pastures have lower WSC concentration, possibly because regrowth increases WSC utilization (Miller et al., 2001). Shade and cloudy conditions can also reduce WSC concentration due to limited photosynthetic activity (Ciavarella et al., 2000; Watts, 2004). Growth stage can influence WSC concentration, which tends to increase from the vegetative to the reproductive stage (Parsons et al., 2004; Jafari, 2012). Forage families, species, and cultivars influence WSC concentration. Satta et al. (2023) found that WSC concentration increases as the percentage of grasses in the pasture relative to legumes increased. Plant ploidy also affects WSC concentration, with typically higher concentrations in tetraploid than diploid cultivars (Rech et al., 2022). Among grass species, WSC concentrations are often highest in modern varieties. As an example, modern perennial ryegrass expressed 25% to 50% higher WSC compared to older varieties (Lee et al., 2001; Miller et al., 2001). In a comparison among C3 species (diploid and tetraploid perennial ryegrass, other C3 grasses, and lucerne) conducted in Denmark, tetraploid ryegrass showed the highest WSC concentration, with 17% and 11.4% of DM in August and October regrowth, respectively (Johansen et al., 2022). Varieties of perennial ryegrass called “high sugars” have been developed to reduce nitrogen excretion in grazing animals and for silage production. In particular, these varieties have been genetically selected for the high-WSC concentration trait, although its expression requires low temperatures (Parsons et al., 2004). However, attempts to increase fructans through genetic modification have not yet led to commercialization of high-fructan lines (Bryan et al., 2025). Few studies tested the ability of annual ryegrass varieties to accumulate WSC (Hopkins et al., 2002). In a recent study comparing 9 cultivars of perennial, hybrid, and Italian ryegrass in New Zealand, the Italian ryegrass (*Lolium multiflorum* Lam.), an annual species, had the highest WSC concentration (up to 22.4%; Sun et al., 2023). High WSC concentrations in grasses are considered typical of high-latitude regions (Van Soest, 1994). In the UK, selected high-sugar grasses showed a range of 20% to 40% WSC concentration (Lee et al., 2001; Miller et al., 2001). However, Rivero et al. (2020) reported average WSC concentrations of 33.2% and 25.9% for high- and low-sugar perennial ryegrass cultivars in south-central Chile, which has a temperate Mediterranean climate.

Similar WSC concentrations have recently been observed in the Mediterranean conditions of Sardinia (Italy) in late winter–early spring. On annual ryegrass, Molle et al. (2022) observed WSC concentrations as high as 29%, and Satta et al. (2023) reported peaks of 36%. Concentrations of WSC between 25% and 30% were found in tetraploid annual ryegrass, with the highest values during cool and sunny days, throughout the period from late February to mid-April (Porcu, 2025). Climate change may favor this trend, as the Mediterranean region has shown an increase in the difference between day and night temperatures (Lionello and Scarascia, 2018) and more frequent winter dry spells, promoting clear skies and low precipitations (Raymond et al., 2018). From a nutritional point of view, the most extensive and detailed literature on pasture WSC relates to equine nutrition. Indeed, WSC, particularly fructans, have been widely studied in grazing horses because overconsumption of high-WSC herbage can induce the onset of pasture-associated laminitis (Schmidt et al., 2023). Although fructans are soluble carbohydrates, they may not be completely digested in the equine foregut, as simple sugars and starch are, but might be fermented in the hindgut as are fibrous structural carbohydrates (Van Soest, 1994). The rapid fermentation of large amounts of WSC may increase the production of volatile organic acids and, in some cases, lactate. This can reduce intestinal pH, leading to hindgut acidosis and associated metabolic disorders such as laminitis, like those caused by excess of starch (Longland and Byrd, 2006). This suggests that the effects of WSC may potentially be similar in ruminants.

However, we cannot assume that hindgut acidosis in horses directly translates to rumen acidosis in ruminants, due to the physiological and fermentation differences between these species. Most studies on grazing ruminants have examined the influence of pasture WSC on feed palatability, intake, and animal production. In contrast, few studies have focused on the influence of increased herbage WSC concentration on rumen function, metabolism, and milk composition. The concentration of WSC is not typically considered when formulating diets for ruminants, especially pasture-fed animals. The effects of WSC, a heterogeneous group of carbohydrates, on rumen fermentation, milk production, and milk composition depend on the source and level of dietary WSC, as well as formulation of the whole diet (Klevenhusen and Zebeli, 2021). Different types of WSC have varying degradation rates and ruminal microbial efficiency (Hall and Weimer, 2016), thus differently affecting rumen pH and VFA profile (Oba, 2011). Unlike simple sugars, fructans are not digestible by mammalian enzymes and are only fermented by ruminal microbes (Hall and Weimer, 2016). Typically, WSC are rapidly degraded by microbes at degradation rates of up to 300% per hour (Sniffen et al., 1992). However, this rate varies greatly among WSC types, depending on factors such as DP (Hall and Weimer, 2016; Klevenhusen and Zebeli, 2021). Different bacteria may degrade long-chain fructans with high DP and short-chain fructans with low DP differently, but research on ruminal fructan degradation is limited (Harlow et al., 2017; Weinert-Nelson et al., 2023). Previous research has focused mainly on simple sugars (monosaccharides and disaccharides) rather than other WSC, such as fructans, or total WSC. Most studies have shown that WSC do not decrease ruminal pH as much as starch does (Oba, 2011). In dairy cows, replacing corn starch with sucrose or molasses (which contains sucrose, glucose, and fructose), at inclusion levels ranging from 2.5% to 7.5% DM, increased or did not affect milk fat (Broderick et al., 2008; Martel et al., 2011). Forages with high WSC can increase palatability, DMI (Jonker et al., 2018), milk production (Miller et al., 2001), and liveweight gain (Lee et al., 2001) in ruminants. This is probably due to changes in the VFA profile produced by WSC fermentation (Ciavarella et al., 2000). Pasture WSC concentration is important for the dietary selection of grazing ruminants (Ciavarella et al., 2000). Diurnal WSC variation is related to the diurnal grazing patterns of cows (Orr et al., 2001), ewes (Molle et al., 2022), and goats (Avondo et al., 2008), showing higher intake or grazing time in the afternoon, when WSC concentrations peak.

Goats grazing in the afternoon had significantly higher milk protein content and lower milk urea content than goats grazing in the morning, probably due to improved ruminal conversion efficiency of pasture N into microbial N at higher WSC concentrations (Avondo et al., 2008). Indeed, a positive association between WSC concentration and N use efficiency has been reported (Ellis et al., 2011; Aizimu et al., 2021), but findings are inconsistent (Thomson et al., 2025). Additionally, Sun et al. (2022) reported a moderate negative relationship between methane yield and the WSC to NDF ratio in sheep fed fresh forages. Lee et al. (2001) reported a strong positive relationship between herbage WSC concentration and liveweight gain in lambs grazing on 2 varieties of perennial ryegrass (*Lolium perenne* L.) with different WSC concentrations. Liveweight increase was attributed to enhanced synchronization of microbial fermentation, increased protein synthesis, and an improved glucogenic-to-lipogenic VFA ratio in the rumen, related to a better balance of energy and nitrogen supply (Lee et al., 2001). However, young grass herbages rich in WSC are often low in CP concentration (often <10% of DM). In fact, WSC and CP concentrations are usually negatively correlated (Rivero et al., 2020; Porcu, 2025), potentially leading to imbalanced energy and protein availability for ruminal microbes. Indeed, Thomson et al. (2025) did not observe improved N use efficiency in lambs fed perennial ryegrass with extreme WSC-to-CP ratios (high WSC: 35.4%, CP: 12%; low WSC: 14.8%, CP: 23.8%). In this study, lambs fed high WSC also produced softer, sweet-smelling, and differently colored feces, likely due to lowered rumen pH and dietary upsets, which also limited microbial protein production. Thus, the effects of WSC on rumen metabolism and animal production might be dose-dependent, with potential negative effects at high doses.

In Sardinia (Italy), lactating dairy ewes grazing on Italian ryegrass (*Lolium multiflorum* Lam.) pasture for 4 h/d in the morning (herbage WSC from 13.5% to 24% of DM) or afternoon (herbage WSC from 16% to 29% of DM), during spring, showed higher post-grazing levels of ruminal propionic and butyric acids, and plasma glucose and insulin, as well as lower blood urea level, in the afternoon than in the morning (Molle et al., 2022). Similarly, castrated male sheep grazing on autumn pasture with a lower WSC concentration (7.7% DM) showed higher acetate-to-propionate ratio, lipogenic VFA level, and pH in the rumen and lower glucose levels in the blood than when grazing on spring pasture with a higher WSC concentration (13.8% DM; Maas et al., 2001). Rivero et al. (2020) reported that in vitro rumen fluid from lactating cows fed perennial ryegrass with high versus medium concentrations of WSC (33.2% and 25.9%) had a lower rumen acetate-to-propionate ratio and reduced methane production. Similar results were obtained in another in vitro study using a basal substrate (25% DM of WSC) derived from fresh perennial ryegrass (*Lolium perenne* L.), at increasing WSC concentrations obtained by adding an 80:20 mixture of inulin and sucrose (Lee et al., 2003). In fact, as WSC levels increased, the glucogenic-to-lipogenic VFA ratio and propionate increased, whereas the acetate-to-propionate proportion decreased linearly and pH decreased. Li et al. (2017) induced laminitis in indoor-fed ewes by infusing a commercial oligofructose (a low-DP fructan of grasses), which resulted in altered rumen fermentation and microbiota and decreased rumen pH. This suggests that increased fructans from grasses might also contribute to a reduction in ruminal pH under certain grazing conditions. Indeed, low rumen pH was reported in Irish dairy cows grazing on a predominantly perennial ryegrass-based pasture, attributed to factors such as high digestibility, high content of rapidly fermentable carbohydrates, and low levels of physically effective fiber in the pasture (O'Grady et al., 2008). The authors suggested that these conditions may contribute to SARA and subsequent health issues in animals (e.g., lameness or laminitis).

Additionally, Molle et al. (2022) found that WSC intake was negatively correlated (r = −0.57) with ruminal pH. In that study, WSC concentrations in the grass varied among days, reaching a maximum value of 29% (Molle et al., 2022). In their review of milk fat depression (**MFD**), Rivero and Anrique (2015) reported that low ruminal pH and MFD can be observed in grazing dairy cows during critical periods, particularly in early spring. This period is characterized by lush pastures in the vegetative stage, with low fiber content, high WSC concentration, and high PUFA content (Rivero and Anrique, 2015). The mechanisms causing MFD in ruminants grazing high-WSC pastures involve alterations in rumen biohydrogenation pathways, driven by PUFA intake from the plants and the production of biohydrogenation intermediates. High WSC can thus contribute to reduced ruminal pH and may interfere with milk synthesis. Altered intake behavior (e.g., increased selection and fast intake rate) and lack of plant fiber effectiveness might act as contributing factors. In Sardinia, Italy, a study of 5 pasture-based dairy sheep farms showed a negative observational association (r = −0.69; *P* < 0.01) between milk fat concentration and pasture WSC concentration, with no association (r ≈ 0) between milk fat concentration and pasture NDF concentration (Satta et al., 2023).

However, these observational associations might have been influenced by changes in NDF, plant maturity, DMI, and rumen dynamics. Recently, Porcu (2025) found that milk fat content was lower in dairy ewes fed unshaded ryegrass herbage with a higher WSC (WSC: 29.2% of DM; milk fat: 5.80%; Figure 1) than in those fed shaded ryegrass herbage with a lower WSC concentration (WSC: 19.2% of DM; milk fat: 6.38%; *P* = 0.004; Figure 1). This reduction in milk fat concentration occurred in the afternoon milking, 3 h after the herbage supply, rather than in the morning milking, the day after the herbage supply. However, despite the observed differences in milk fat concentration, fat yield did not differ significantly between the 2 groups, suggesting a possible dilution effect. In the same study, milk fat concentration of the afternoon milking showed a negative observational association with daily WSC intake (R^2^ = 0.81; *P* < 0.01, using mean values per group for each sampling day) but not with daily NDF intake. Furthermore, PUFA levels did not differ between shaded and unshaded grass (fat: 2.7% and 2.5% of DM; PUFA: 73.8 and 74.3 g/100 g total fatty acid methyl esters, respectively), indicating that the lower milk fat concentration in the ewes fed unshaded herbage was not due to excess of PUFA. In a follow-up trial on ewes fed high-WSC herbage (WSC: 28%), Porcu (2025) found that a single dose of 25 g/d of sodium bicarbonate, supplied half an hour before grass herbage was supplied (4 h before afternoon milking), improved total DMI, herbage intake, milk fat percentage (*P* < 0.001; Figure 1), and milk fat yield. These results suggest that buffer supplementation may prevent ruminal pH reduction, which in turn might avoid the reduction in milk fat concentration associated with high-WSC grass intake in dairy ewes. However, Rearte et al. (1984) reported that the use of buffers (1.9% of the concentrate, i.e., on average 185 g/d) failed to alleviate moderate MFD associated with the use of high-quality grasses and clover mix in grazing dairy cows. Unfortunately, WSC concentrations were not reported in that study. In conclusion, the concentration of WSC in grasses is highly variable and strongly depends on environmental and management factors, as well as grass species, variety, and type.

**Figure 1:**
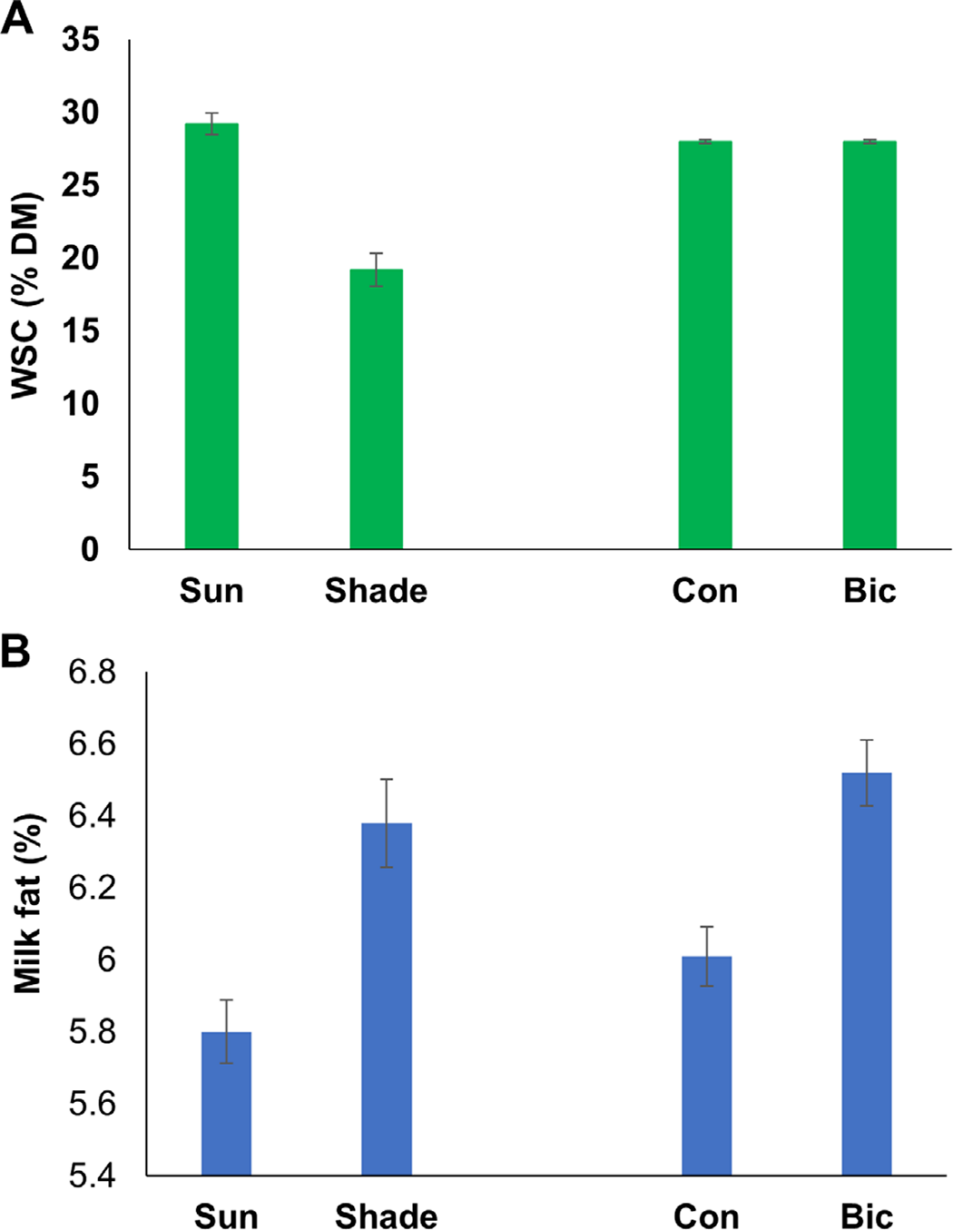
(A) Water-soluble carbohydrate (WSC) concentrations in a study on dairy ewes fed unshaded (Sun) or shaded (Shade) annual ryegrass herbage indoors (mean and SE of the last 4 sampling days, when the WSC difference between treatments was the greatest), and in a follow-up study where ewes were fed high-WSC annual ryegrass indoors with 25 g/d of sodium bicarbonate supplementation (Bic) or without it (control; Con). (B) The corresponding covariate-adjusted means and SE of milk fat concentration (%) in afternoon milkings, performed 3 h (first study) or 4 h (second study) after herbage supply (modified from Porcu, 2025).

Thus, frequent monitoring of herbage composition is required for optimal pasture management in grazing ruminants. Pasture WSC favors efficient pasture N utilization, but high WSC intake may negatively contribute to changes in rumen fermentation and milk fat synthesis under certain grazing conditions. However, the mechanisms involved remain unclear and are likely influenced by multiple dietary factors. The optimal WSC concentration in the diet of grazing ruminants has not been defined yet and depends on several factors, including the combination of pasture with other feeds. Further studies are needed to better understand the mechanisms underlying the effects of grass WSC on rumen function and milk composition, particularly milk fat concentration. In addition, studies are needed to determine the optimal inclusion level of WSC in the diets and proper feeding strategies for ruminants grazing pasture rich in WSC.
